# Determining Which Cooking Method Provides the Best Sensory Differentiation of Potatoes †

**DOI:** 10.3390/foods9040451

**Published:** 2020-04-07

**Authors:** Melissa Ciccone, Delores Chambers, Edgar Chambers IV, Martin Talavera

**Affiliations:** 1 Center for Sensory Analysis and Consumer Behavior, 1310 Research Park Dr., Manhattan, KS 66502, USA; mciccone@ksu.edu (M.C.);; 2 Center for Sensory Analysis and Consumer Behavior, Kansas State University, Olathe, KS 66061, USA; talavera@ksu.edu

**Keywords:** potatoes, sensory, cooking method, high identity traits, HITS

## Abstract

There are many ways to prepare potatoes that each provide a unique set of sensory properties. However, when conducting a descriptive sensory study, it is important to utilize a cooking method that will highlight, and not distract from, the sensory differences among potato samples due to factors such as variety or growing conditions. This study aimed to determine which of five cooking methods results in the best differentiation among potato varieties to recommend a single method for use in future descriptive sensory studies. Five different potato varieties were each prepared using boiling, mashing, baking, frying, and air frying methods. The samples were provided to six highly trained descriptive panelists and evaluated by consensus using a modified high identity traits (HITs) method. Panelists evaluated the aroma, flavor, and texture to develop a list of up to five total HITs per sample. Additionally, panelists scored each sample for degree of difference (DOD) from the control. Based on the HITs profiles and DOD scores, mashing, baking, and air frying methods were all effective in differentiating the samples. Frying and boiling methods introduced too much variation and are not recommended for sample differentiation. Ultimately, the method chosen for future research would depend on the study objectives.

## 1. Introduction

Potatoes are the most consumed vegetable in the United States, with a per capita value in 2016 of about 21 kg (46 lbs), with almost half of the consumption coming in the form of fresh potatoes. This is 7 kg (16 lbs) more than the per capita consumption of the second vegetable on the list, tomatoes [1]. Additionally, according to the National Potato Council, the United States ranks as one of the world’s top five producers of potatoes, coming in at just over 20 million tons in 2017 [2]. With high rates of both production and consumption, potatoes are likely to remain a staple of American diets for many years to come.

With over 200 varieties sold in the U.S., there are a wide range of textures, flavors, and aromas that exist among potatoes [3]. Descriptive sensory analysis can be utilized to help measure the individual texture, flavor, and aroma attributes of each of the potato varieties to create complete sensory profiles. This allows for the categorization of varieties into groups with similar characteristics, which can provide a lot of valuable information to both producers and consumers. Previous research has been done to determine what factors affect the sensory properties of potatoes, but much of the research has focused on defects or off-flavors that can potentially affect potato quality or consumer health [4,5]. Another large category of potato sensory studies involves relating sensory data to analytical measurements such as moisture content or hardness [6,7,8]. There are fewer studies that have explored differences in sensory properties across varieties, but Jansky [9] compared the sensory properties of baked potatoes for thirteen different cultivars. Rizzo et al. [10] examined six varieties using *sous vide* cooking and found differences in browning, “soil odor,” and sweetness among the freshly prepared varieties. Sharma et al. [11] examined consumer acceptance of mashed potatoes for 12 cultivars and showed that there were segments of consumers who liked certain potatoes more. However, those authors did highlight certain cultivars that were liked by all segments and others that were disliked by all segments. Other authors [12] studied a few sensory properties of multiple cultivars of potatoes using several different cooking methods and concluded that differences existed in sensory properties. Unfortunately, the attributes were measured inconsistently across cooking methods, and some of the cooking methods used additives such as milk, butter, or salt, which may have masked some flavors or textures.

While the highlighted research provides valuable insight into the sensory properties of potatoes, most of these studies focused on preparing the samples with only one cooking method. Potatoes are an extremely versatile food and can be prepared in many ways, making the sensory characterization of potatoes much more complex because the sensory properties can be reliant on the cooking method. Cooked potato preparations can include dry heat transfer methods such as baking, roasting, deep frying, or air frying. These methods are generally accompanied by reactions such as Maillard browning and caramelization, which can affect the exterior texture by making it crisp or affect the flavor by imparting toasted, bitter, or sweet attributes. Additionally, some of these methods involve the use of oil, such as olive or canola, which can draw attention away from the natural flavors or aromas of the potato. Methods such as boiling, mashing, and steaming are moist heat cooking preparations, and the use of water can add moistness but may also create an accumulation of starch which can affect the sensory properties.

Because different cooking methods can lead to different sensory profiles, it is important to consider multiple cooking methods when evaluating potato samples [12]. There have been previous studies that looked at the effect of different cooking methods of potatoes on sensory properties but stayed within a single sample form. For example, Giovanelli et al. [13] compared appearance, aroma, flavor, and texture attributes for French fries using deep-fat frying, air frying, baking, and microwaving. Another study also evaluated full sensory profiles for deep fried French fries versus air fried French fries [14]. Seefeldt et al. [15] compared twenty-six different attributes across three different cooking methods and forms: boiled, mashed, and oven fried.

When evaluating the sensory profiles of different varieties of a product, it is important to keep all other factors the same, so that any differences found in sensory properties can be attributed to true differences among the samples [16]. Although many possible preparation methods are possible for many products, it usually is necessary to choose only one or two methods for most general purpose products to reduce the time and money needed to conduct the research and make decisions. The decision about which preparation method(s) to use may need to be facilitated by previous research or a preliminary study to test the options. Examples of preliminary studies have been carried out using products such as coffee and green tea [17,18]. There have not been any previous studies conducted to determine the best cooking method for differentiation of potatoes.

The objectives of this study were to determine which cooking method(s) would result in the most differences in sensory properties of potato varieties themselves, and to recommend one or more cooking method(s) for use in future studies comparing the properties of different potato cultivars.

## 2. Materials and Methods

### 2.1. Potato Samples

For this study, five varieties of raw potatoes were purchased from a local grocery store in Manhattan, Kansas. The five varieties were Red, Gold, Petite White, Russet Norkotah, and Russet Burbank. These varieties were chosen for their commercial availability, consumption popularity, and range of sensory properties. Two varieties of Russet potatoes were included to test the sensory method’s ability to differentiate between similar samples. After purchase, samples were kept in a cool, dark room in their original packaging. All samples were used within ten days of purchase.

### 2.2. Sample Preparation

Five cooking methods (Table 1) were chosen to represent common commercial and home preparation methods for potatoes. The methods cover both moist and dry heat preparations. The following procedures were followed as written for the Red, Gold, Russet Norkotah, and Russet Burbank varieties. Note that due to the small size of the Petite White potatoes, if the skin was removed prior to cooking, it was done with a knife instead of a peeler. Boiled, mashed, and baked samples were done cooking when a fork could be easily inserted through the center of the sample, meaning the potato was tender all the way through. A similar method for determining doneness was also used by Tian et al. [19]. Water treated by reverse osmosis and carbon filtration was used to rinse and cook samples as needed. The use of ultra-purified water to help prevent additional substances present in water from affecting the properties of the final samples was suggested by Nguyen do Trong et al. [20].

#### 2.2.1. Boiled

The skin of each potato was removed using a vegetable peeler, then each potato was rinsed under deionized water to remove any remaining dirt. Each potato was cut into slices using a Vollrath Redco^®^ InstaCut™ 5.0 countertop food slicer (The Vollrath Company, LLC, Sheboygan, WI, USA) fit with a 1.3 cm (½ in.) stainless steel dicing blade, then the slices were cut down to ½” cubes using a knife. In a large saucepan, 225 g of ½” cubes were covered with 600 mL of deionized water. The saucepan was covered with a lid and brought to a boil on a gas stovetop. Once boiling, the heat was reduced to a medium simmer and the potato cubes were cooked until a fork could be easily inserted into the center. The pan was then removed from the heat and the water was drained from the cooked cubes. The boiling method was slightly adapted from the procedure followed by Oruna-Concha et al. [21].

#### 2.2.2. Mashed

The potatoes were prepared and diced into ½” cubes according to the method used for the boiled potato samples. In a large saucepan, 450 g of ½” cubes were covered with 800 mL of deionized water. The saucepan was covered with a lid and brought to a boil on a gas stovetop. Once boiling, the heat was reduced to a medium simmer and the potato cubes were cooked until a fork could be easily inserted into the center. The pan was then removed from the heat and the cooked cubes were drained, reserving the cooking water. The cooked potatoes were pressed through a potato ricer (OXO, New York City, NY, USA) into a bowl. Next, 35 mL of the reserved cooking water was added and the potatoes were stirred with a fork until smooth and consistent in texture.

#### 2.2.3. Baked

Potatoes of similar size were chosen for each variety. The outside of each potato was scrubbed with a sponge to remove dirt from the skin, then each potato was thoroughly dried with a clean cloth. Two opposite sides of each potato were punctured ½” deep to allow for steam to escape. Next, the potatoes were individually wrapped in foil. All potatoes of the same variety were placed on a single baking sheet and baked at 425 °F (218 °C) until a fork could be easily inserted into the center. The potatoes were removed from the oven, left to cool slightly, and then unwrapped. The flesh of each potato was scooped out of the skin using a spoon and cut into ½” cubes.

#### 2.2.4. Fried

The skin of each potato was removed using a vegetable peeler, then each potato was rinsed under deionized water to remove any remaining dirt. Each potato was cut into slices using a countertop food slicer fit with a ½” stainless steel dicing blade. Using only slices that were cleanly cut on all four sides, the slices were cut into 2” long pieces with squared off edges. The slices were soaked in deionized water for 30 min at room temperature. After soaking, the pieces were drained and the surface of each slice was dried with paper towels. Canola oil was heated in a 6-quart countertop electric deep fryer (National Presto Industries, Inc., Eau Claire, WI, USA) to 275 °F (135 °C), and the potato slices were fried in batches of 15 pieces for 5 min. After the pieces were removed and drained, the temperature of the oil was increased to 350 °F (176 °C), and the slices were fried for a second time until golden brown in color. No more than two samples were fried in a single batch of oil to prevent too much flavor transfer between samples.

#### 2.2.5. Air Fried

The potatoes were prepared, cut, and soaked according to the method used for deep frying. After the slices were removed from soaking and dried, they were evenly coated with 5 mL of canola oil. The coated slices were arranged with even spacing on a copper-coated crisping tray and baked in a 425 °F (218 °C) oven until golden brown in color.

### 2.3. Sensory Evaluation

The potato samples were evaluated by six highly trained panelists from the Center for Sensory Analysis and Consumer Behavior at Kansas State University in Manhattan, KS, USA (Institutional Review Board #9384). The panelists all received a minimum of 120 h of general descriptive sensory analysis training prior to completing this panel. The first two days of the panel consisted of 90-min orientation sessions to introduce the method and familiarize the panelists with the types of samples they would be evaluating. Then, the panel completed five days of evaluation, with one type of cooking method being presented each day. Similar panels have been used by other authors for food products [16,22,23,24,25,26,27,28,29,30,31] and chemical compounds [32,33].

Samples were labeled with random three-digit blinding codes and were served one at a time according to a Latin Square design, so that each potato variety appeared in each serving position once over the five days. The samples were served in the following amounts: 8–10 cubes for boiled and baked methods, 65 g for the mashed method, and 3 pieces for deep fried and air fried methods. All samples were served in 4 oz glass jars covered with watch glasses. The glass jars were placed on top of heated tiles in metal trays to maintain a serving temperature range of 40–45 °C.

#### 2.3.1. High Identity Traits

The sensory profiles of the potato samples were determined using a modified high identity traits (HITs) method, which can be used to develop shorter, more simplified lists of terms to describe products [34]. The simplistic nature of the profiles allows for more rapid evaluation of products. The panelists were instructed to develop a list of no more than five traits (HITs) to describe aspects of the aroma, flavor, and texture that were most important to the identity of each sample. After tasting each sample, the panelists decided on a consensus list of traits through group discussion. Then, the panelists used a consensus evaluation to rate the intensity of each HIT on a scale from 0–15 with 0.5 increments, where 0 means the trait is not present and 15 represents an extremely high intensity. This was different from the original Talavera et al. [34] study, which only rated the intensities as slight, moderate, or strong. The more complex scale used in this study potentially allows for more differentiation among samples with the same HIT. Group discussion was also used to establish a definition for each HIT identified. There were no references used during the evaluation of the samples.

#### 2.3.2. Degree of Difference

Panelists also evaluated the degree of difference (DOD) from the control [35] for each sample. In addition to the five varietal samples served each day, panelists were blindly served a second Red potato sample labeled as the control. After developing the HITs profiles for each sample, the panelists rated how different the sample was from the control sample based on aroma, flavor, and texture. After group discussion, the DOD was rated by consensus on a scale from 0–15 with 0.5 increments, where 0 represents no perceived difference and 15 represents the greatest possible difference. The DOD scale was treated as product-specific, so any perceived differences were only rated against other varieties of potatoes.

### 2.4. Data Analysis

All data analysis was performed using Excel 2016 (Microsoft Corporation, Redmond, WA, USA) and XLSTAT 2018 (ADDINSOFT, New York, NY, USA). Bar charts were used to display HITs intensity and DOD scores. Principal component analyses were run using the covariance matrices for each cooking method and for all samples combined. Zeros were used to fill in intensity scores for traits that were not present in samples to provide a complete matrix for analysis. These methods are recommended or have been used for similar studies [36,37,38,39].

## 3. Results

### 3.1. High Identity Traits

The panel used a total of twenty-four terms to describe the high identity traits (HITs) profiles of the samples across all five cooking methods. The list included five terms for aroma, eleven for flavor, and eight for texture. All traits and their definitions can be found in Table 2. Out of the twenty-four traits, only potato flavor was used to profile all samples for all cooking methods.

Some of the cooking methods produced samples with traits that were unique to that preparation. For example, starchy flavor was only used to describe the mashed and boiled samples. Despite having a trait common to several samples, the boiled and mashed cooking methods still produced the highest numbers of differentiating traits. These are traits that were used to describe only one sample within the cooking method, and both methods had five differentiating traits.

**Figure 1:**
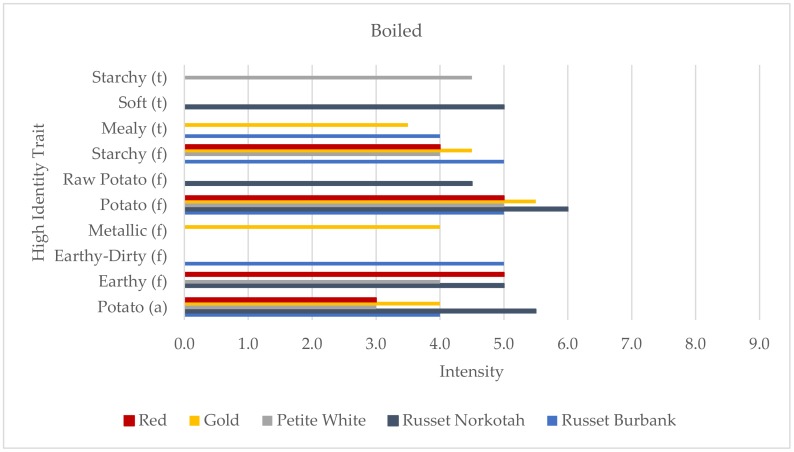
High identity traits (HITs) profiles of the boiled potato samples (aroma (a) the “smell” of the product; flavor (f), tastes, aromatics, and feeling factors while food is being eaten by the mouth; and texture (t)). Intensity was rated on a 0–15 scale with 0.5 increments.

The differentiating traits for the boiled samples included starchy texture, soft texture, raw potato flavor, metallic flavor, and earthy-dirty flavor (Figure 1). Starchy texture was profiled for the Petite White sample, soft texture and raw potato flavor were only used for the Russet Norkotah sample, metallic flavor was differentiating for the Gold sample, and earthy-dirty flavor was unique to the identity of the Russet Burbank sample. A total of ten traits were used to describe the boiled potato samples, including three traits for texture, six for flavor, and only one for aroma. While there was a high number of differentiating traits, two traits, potato aroma and potato flavor, were used to profile all five boiled samples. Russet Norkotah was scored as having the highest intensity for both. The Red sample was the only variety with a HITs profile of fewer than five traits, and was described using only four because the panelists could not agree on a fifth trait and ultimately decided that the sample’s sensory profile was simpler than the other varieties. It was also the only sample with a HITs profile that did not contain any of the three texture terms, starchy, soft, or mealy.

**Figure 2:**
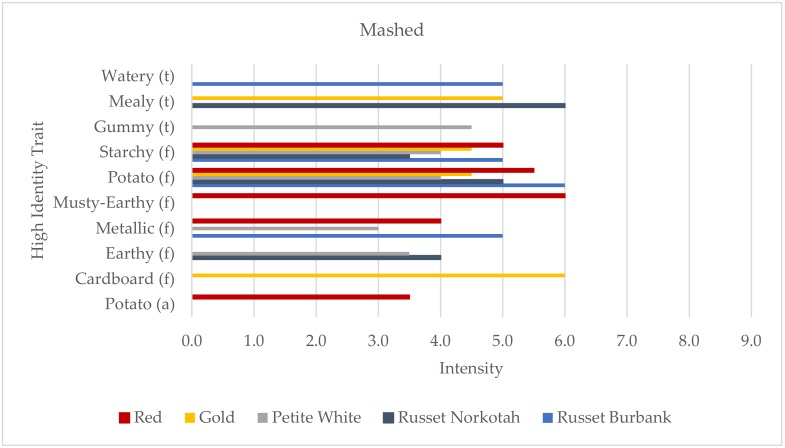
High identity traits (HITs) profiles of the mashed potato samples (aroma (a), the “smell” of the product; flavor (f), tastes, aromatics, and feeling factors while food is being eaten by the mouth; and texture (t)). Intensity was rated on a 0–15 scale with 0.5 increments.

The mashed potato samples were also profiled using a total of ten HITs, with three traits for texture, six for flavor, and one for aroma (Figure 2). The mashed cooking method was the only one that produced more than one sample that was profiled using fewer than five traits. The profiles of the Gold, Russet Norkotah, and Russet Burbank samples all contained only four HITs. Starchy flavor and potato flavor were the only two traits out of the ten that were used to profile all five of the mashed samples, with the Red and Russet Burbank samples receiving the highest intensity scores for both. Unlike the boiled cooking method, potato aroma was only present in one sample, which was the Red variety. The Red sample was also differentiated from the other samples by musty-earthy flavor. Other differentiating traits included watery texture, which was only included in the Russet Burbank profile; gummy texture, which was unique to the Petite White profile; and cardboard flavor, which was only present for the Gold sample.

**Figure 3:**
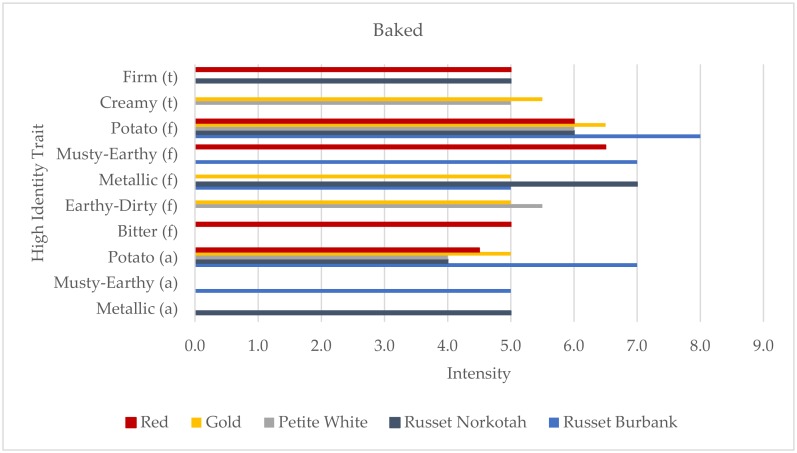
High identity traits (HITs) profiles of the baked potato samples (aroma (a) the “smell” of the product; flavor (f), tastes, aromatics, and feeling factors while food is being eaten by the mouth; and texture (t)). Intensity was rated on a 0–15 scale with 0.5 increments.

A total of ten traits were used to describe the five baked potato samples. The traits included two traits for texture, five for flavor, and three for aroma (Figure 3). The Petite White sample was the only variety with a HITs profile of fewer than five traits and was described using only four. Potato aroma and potato flavor were used to profile all five varieties, but Russet Burbank scored highest in intensity for both. Russet Burbank was also the only baked sample that was not profiled using either of the texture traits, firm or creamy. Instead, the profile focused on aroma and flavor traits, also including musty-earthy flavor and aroma and metallic flavor. Traits that were only used to profile one variety included bitter flavor, musty-earthy aroma, and metallic aroma. Bitter flavor was profiled in the Red sample, musty-earthy aroma was used to describe the Russet Burbank sample, and metallic aroma was important to the identity of the baked Russet Norkotah sample.

**Figure 4:**
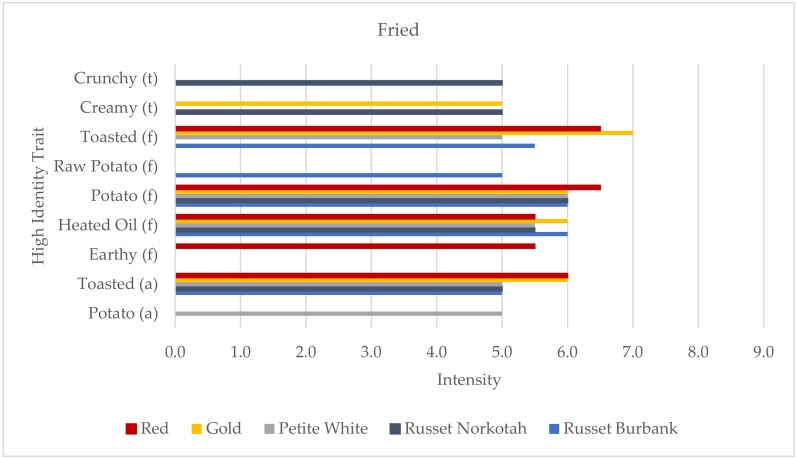
High identity traits (HITs) profiles of the fried potato samples (aroma (a) the “smell” of the product; flavor (f), tastes, aromatics, and feeling factors while food is being eaten by the mouth; and texture (t)). Intensity was rated on a 0–15 scale with 0.5 increments.

The nine overall traits for the fried samples included two for texture, five for flavor, and two for aroma (Figure 4). All five samples were profiled using the maximum of five HITs, although potato flavor, heated oil flavor, and toasted aroma were all used as traits for all varieties. However, there were still four differentiating traits: crunchy texture, raw potato flavor, earthy flavor, and potato aroma. Crunchy texture was unique to the Russet Norkotah sample, raw potato flavor to the Russet Burbank, earthy flavor to the Red, and potato aroma to the Petite White.

**Figure 5:**
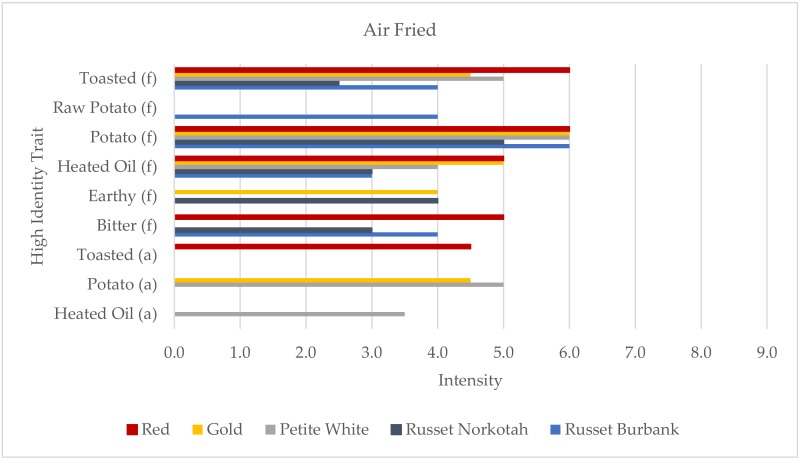
High identity traits (HITs) profiles of the air fried potato samples (aroma (a) the “smell” of the product; flavor (f), tastes, aromatics, and feeling factors while food is being eaten by the mouth; and texture (t)). Intensity was rated on a 0–15 scale with 0.5 increments.

The five air fried samples were also profiled using only nine traits, with three for aroma and six for flavor (Figure 5). This was the only cooking method where texture terms were not included in the HITs profiles. Like the fried samples, all five of the air fried samples were profiled using the maximum of five traits, but three of the traits were the same across the samples: potato flavor, toasted flavor, and heated oil flavor. Again, the profiles were dominated by oil and browning-related traits, so only three of the samples had differentiating traits. Raw potato flavor was unique to Russet Burbank, toasted aroma to Red, and heated oil aroma to Petite White.

**Figure 6:**
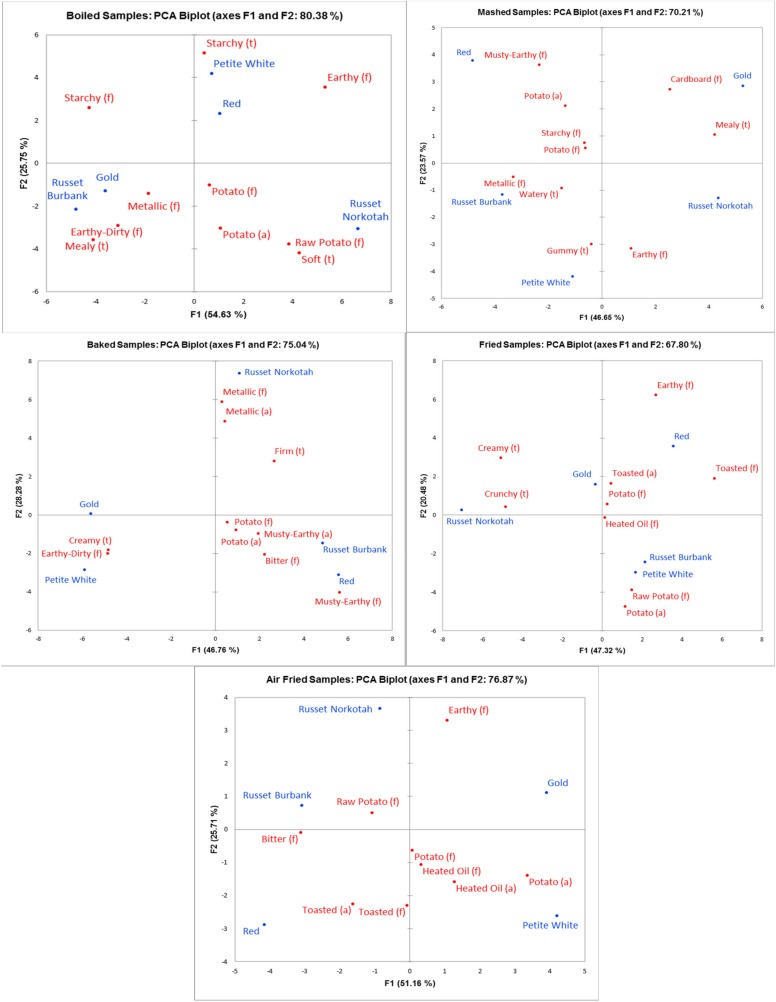
Principal component analyses (PCA) by cooking method of aroma (a), flavor (f), and texture (t) HITs for all potato varieties.

The Principal Components Analysis (PCA) bi-plots for the individual cooking methods are shown in Figure 6. The fried and air fried samples were dominated by heated oil and toasted properties, so the samples lie closer to the center of their respective bi-plots than the samples for each of the other three methods. However, despite the lower number of differentiating traits, the air fried samples appear more differentiated across their bi-plot than the fried samples. The mashed samples are well-differentiated because all samples lie close to the edge of the plot, with each sample appearing in its own space.

### 3.2. Degree of Difference

**Figure 7:**
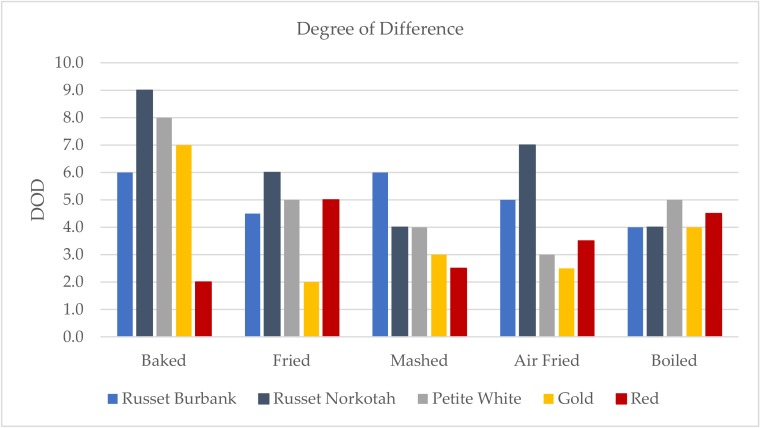
Degree of difference (DOD) from control scores for all varieties and cooking methods. DOD was rated on a 0–15 scale with 0.5 increments.

As shown in Figure 7, the fried Red sample produced the highest DOD score from the control out of the Red potato samples. The boiled Red sample also produced a high DOD score, which was not expected since the control sample was also the Red variety. The high scores could have been due to increased variability among the samples based on preparation method.

**Figure 8:**
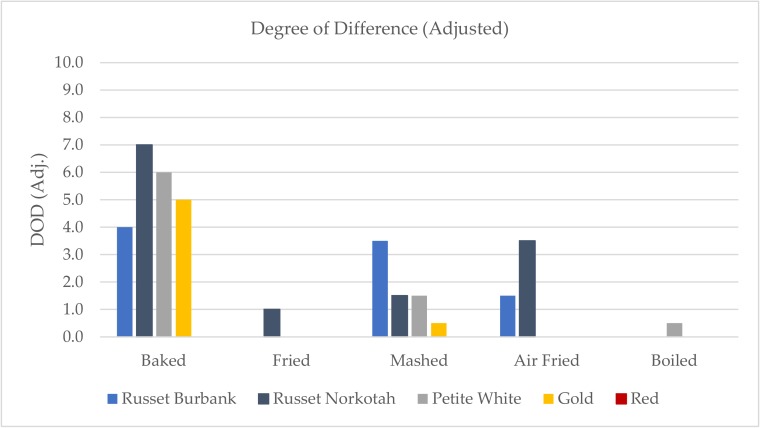
Adjusted degree of difference (DOD) from control scores for all varieties and cooking methods. DOD was rated on a 0–15 scale with 0.5 increments.

Since the Red samples showed that the DOD scores may be affected by preparation variation, all of the DOD scores were adjusted to account for that. The Red sample DOD scores were set to zero to represent that they were equal to the control sample. Any samples with DOD scores lower than the Red sample were also set to zero because they were not found to have more differences from the control than the Red sample. Varieties with higher DOD scores were adjusted down proportionally to their original differences with the Red samples. The adjusted DOD scores are shown in Figure 8, and the remaining results will focus on these scores.

Baking and mashing were the only two cooking methods within which all four non-Red varieties exhibited differences from the control, with the baked samples producing the largest differences. Out of the baked samples, the Russet Norkotah received the highest DOD score. Looking at the HITs profile for the baked Russet Norkotah sample, it was the only sample scored for metallic aroma and also received a high intensity score for metallic flavor, so these characteristics may have contributed to the DOD. Russet Burbank received the highest DOD score of the mashed samples. This was mostly because of the watery texture that was unique to only that sample.

The fried and boiled methods produced the least amount of differences from the control sample. Both methods had only one sample that was scored differently from the control after adjustments, and the DOD scores for the single sample were 1.0 or below for both preparations.

## 4. Discussion

Despite only focusing on five high identity traits per sample, there were sensory differences identified among the samples. This can be concluded because, out of twenty-four total high identity traits (HITs) used to describe the samples, only potato flavor was used to profile all samples across all five cooking methods. That is similar to prior authors [12], who found different attributes for each of the potato cooking methods they studied. The differences in HITs terms for all samples allows for comparison of the numbers and types of traits used for each sample to establish which cooking methods are best for potato differentiation.

In addition to the overall HITs differences, some of the cooking methods produced samples with traits that were unique to that preparation. For example, starchy flavor was only used to describe the mashed and boiled samples. Higher intensities of starchy traits are expected in moist heat cooking methods such as mashing and boiling because the water causes the potatoes to release starch as the starch granules swell and burst. Rizzo et al. [10] found “floury” characteristics in potatoes cooked using a *sous vide* moist heat method, which may be related to the starchy attribute found by our panel. Even with having a trait common to several samples, the boiled and mashed cooking methods still produced the highest numbers of differentiating traits, so these two methods can effectively highlight differences among potato varieties.

The mashed cooking method was the only one that produced more than one sample that was profiled using fewer than five traits. The profiles of the Gold, Russet Norkotah, and Russet Burbank samples all contained only four HITs, suggesting that the mashed cooking method may produce more simple sensory profiles than the other cooking methods. This could be because the mashing of the samples creates a more homogenous texture, and the addition of cooking water in the method may dilute the aroma and flavor. One interesting feature of the mashed samples was the differentiating trait of cardboard flavor present in the Gold sample because, according to a study done by Blanda et al., cardboard is generally considered an off-flavor or defect [4]. It is possible that the Gold potatoes used for mashing were not the highest quality, which further highlights the possibility of high variability within the sample since quality can differ from potato to potato.

Like the boiled and mashed methods, frying and air frying also had method-specific traits. Toasted and heated oil aromas and flavors were only used to describe samples cooked using the fried and air fried methods. These were the only two methods that called for oil as part of the procedure, so it was expected that the oil would impart certain aromas and flavors. Additionally, frying and air frying are both dry heat cooking methods, so browning reactions are more likely to occur and create toasted sensory properties. While frying and air frying were differentiated from the other cooking methods by heated oil and toasted traits, they decreased the ability to highlight sample differences within the methods by masking other aroma and flavor properties. Boiled, mashed, and baked samples were each described using a total of ten traits, but the fried and air fried samples were each only described using nine.

Air frying was the only cooking method where texture terms were not included in the HITs profiles. This is most likely because the exterior texture of the samples was unique but also inconsistent due to the cooking method. Since the texture was new to most of the panelists, they could not determine if the texture of each sample was due to the cooking method or varietal differences, so they avoided using texture terms altogether. A 2015 panel conducted by Teruel et al., also found that air fried samples had a unique texture with a puffy and dry exterior appearance different from their deep-fried counterparts [14].

By comparing the PCA bi-plots for the individual cooking methods, some insight can be gained about the ability of each method to differentiate samples (Figure 6). As previously mentioned, the fried and air fried samples were dominated by heated oil and toasted properties, but the air fried samples were more differentiated than the fried samples. This is most likely because the air fried samples showed more differences in intensity for the traits that were shared by all five samples, allowing for more separation on the map. Ultimately, neither frying method appears to be great for sample differentiation, but the air fried method could be somewhat more effective if a comparison of browning effects across varieties is desired.

Based on the PCA bi-plot alone, mashing appears to be the best method for sample differentiation. This is interesting because the mashed method produced the most samples with four-term profiles, so it would seem like the homogenization of the samples by mashing would dilute the sensory properties and prevent differentiation. This result is also contradictory to a previous study that found less variation among mashed samples compared to boiled and oven-fried samples, and concluded that the lack of variation was due to the mashing homogenizing the sample [15]. However, the results for the present study showed that the mashed samples were still profiled using a high number of overall HITs and the highest number of differentiating traits, and the mashed method is capable of differentiation.

The addition of degree of difference (DOD) scores can help provide more information about the differentiation of samples than the HITs profiles alone. This is because panelists are considering all aspects of aroma, flavor, and texture at once and comparing them to a control sample. Since the control sample was the same variety as one of the evaluated samples, the DOD score for the Red sample each day can give insight into a few different experimental factors. Some degree of difference between the control sample and the evaluated Red sample is acceptable as it may be a result of natural variation within the potato variety. However, DOD scores greater than 2.0 may suggest either inconsistencies with sample preparation or an issue with panel calibration. Since the panel used for this study was highly trained, any higher DOD scores for the Red potato samples were most likely due to problems with sample preparation consistency for some of the cooking methods. The control sample was served at the beginning of the panel every day, but due to sample randomization, the Red evaluated sample was always in a different serving position. Therefore, the control sample and Red sample were prepared separately to allow for consistency in the time between cooking and serving.

Despite careful procedural considerations to ensure sample consistency, the fried and boiled Red samples both produced higher DOD scores than expected. This was most likely due to the variable nature of these two cooking methods. Frying is a difficult process to control because the oil temperature can be hard to regulate, the samples may cook unevenly in different parts of the fryer, and sample texture can change drastically with increased time between cooking and serving. The sample for the boiled preparation was cut into small cubes, so it is possible that within sample variation was more noticeable if the texture or flavor was different from cube to cube. Additionally, despite frequent stirring, the cubes may have cooked unevenly during boiling based on their locations in the pot, leading to inconsistent texture. These inconsistencies led to the decision to adjust the DOD scores to account for variability between the control samples and the Red samples based on cooking method to allow for differences in sensory properties based on variety to be highlighted.

Overall, the baked samples produced the largest differences from the control. The baking method was the only one that did not directly introduce water or oil to the sample, so the lack of interference may allow for more of the varietal differences to stand out. Mashed samples also exhibited differences from the control for all samples. The mashed method may be the most resistant to sample preparation variation because inconsistencies in texture due to cooking are ultimately homogenized during mashing. The fried and boiled methods produced the least amount of differences from the control sample. Frying and boiling both introduce a factor into the preparation that is not present in the natural samples (oil and water, respectively), so the added noise may dilute any differences in the samples due to potato variety.

## 5. Conclusions

The combination of high identity traits profiles and degree of difference scores proved to be useful in conducting this sensory study. The HITs profiles can help compile quick and simple information about the sensory modalities of interest which can begin highlighting characteristic differences among samples. While the range of HITs used and the number of differentiating traits can begin to show how well a cooking method can highlight differences among samples, the DOD scores provided the most information about differentiation and were a valuable addition to the methods of this study.

Based on the results of this study, the cooking methods that are most useful in differentiating potato varieties are mashing and baking. One or both of these two methods is recommended for use in future sensory studies to compile more information about or compare potato samples, such as a lexicon development study. If the objective of a study is to determine differences in potato flavor, mashing is recommended because it was found to homogenize differences in the sample texture due to preparation inconsistencies. This allowed the panel to focus more on flavor characteristics. If the objective of a study is to evaluate the full sensory profiles of potato samples, then baking is recommended because it is the only cooking method tested that does not introduce additional noise in the form of water, oil, or texture manipulation. It is worth noting that neither mashing nor baking allow the potatoes to participate in browning reactions, so if a key objective of a study is to observe sensory differences based on those reactions, then an air fried cooking method is recommended. Air frying is recommended over frying because it is an easier process to control that could provide more consistent results.

Whichever method is chosen to cook potato samples, it is important to keep in mind that consistency with the preparation procedure is key to ensuring that any differences found among samples are as unaffected by external noise and sources of error as possible. A specific procedure should be developed and strictly followed, with special attention paid to sample shape, sample size, cooking time, and cooking temperature.

## Figures and Tables

**Table 1:** Approximate cooking times per method for each potato variety.

	Boiled	Baked	Fried	Mashed	Air Fried
**Red**	19 min	50 min	5 1/2 min	19 min	32 min
**Russet Burbank**	13 min	45 min	6 min	13 min	30 min
**Russet Norkotah**	15 min	45 min	6 1/2 min	15 min	30 min
**Gold**	21 min	50 min	6 min	21 min	32 min
**Petite White**	23 min	35 min	5 1/2 min	23 min	32 min

**Table 2:** Definitions of high identity traits (HITs) used for evaluation of potato samples.

	High Identity Trait	Definition
**Aroma**	Heated Oil	The aromatics associated with oil that has been heated.
	Metallic	An aromatic associated with tin cans or aluminum foil.
	Musty-Earthy	The musty aromatics associated with raw potatoes, decaying vegetation, and damp soil.
	Potato	The starchy and slightly metallic cooked vegetable characteristic associated with the meat of the potato.
	Toasted	A moderately brown, baked impression.
**Flavor**	Bitter	A fundamental taste sensation that is characterized as being acrid, sharp, or pungent. May include a lingering flat taste over the back of the tongue.
	Cardboard	The aromatic associated with cardboard or paper packaging.
	Earthy	An aromatic that has a damp, earthy character similar to fresh mushrooms or raw potato.
	Earthy-Dirty	Dry, dirt-like aromatic associated with dry soil.
	Heated Oil	The aromatics associated with oil that has been heated.
	Metallic	An aromatic and mouth feel associated with tin cans or aluminum foil.
	Musty-Earthy	The musty aromatics associated with raw potatoes, decaying vegetation, and damp soil.
	Potato	The starchy and slightly metallic cooked vegetable characteristic associated with the meat of the potato.
	Raw Potato	The starchy, raw vegetable-like character associated with peeled, sliced, uncooked potatoes. May include slight green or unripened notes.
	Starchy	The aromatics associated with starch and starch-based vegetables such as corn, potatoes, and legumes.
	Toasted	A moderately brown, baked impression.
**Texture**	Creamy	The rich, smooth, full feeling in the mouth which may be thick and slick.
	Crunchy	The force and noise with which the sample breaks, cracks, or ruptures.
	Firm	Requiring a moderate amount of force to bite completely through the sample.
	Gummy	A sticky, gluey impression perceived in product during mastication.
	Mealy	The perception of fine, soft, somewhat rounded smooth particles very evenly distributed within the product itself.
	Soft	The lack of resistance to the teeth when biting down on the product.
	Starchy	Degree to which the sample mixes with saliva to form a starchy, pasty slurry that coats mouth surfaces after swallowing.
	Watery	The perceived amount of moisture in the product when placed in the mouth.
